# Effects of Hydrofluoric Acid Concentration and Etching Time on the Bond Strength to Ceramic-coated Zirconia

**DOI:** 10.3290/j.jad.b2838165

**Published:** 2022-03-24

**Authors:** Chunxiao Jin, Jingrong Wang, Yutian Huang, Ping Yu, Yuhuan Xiong, Haiyang Yu, Shanshan Gao

**Affiliations:** a Postgraduate Student, State Key Laboratory of Oral Diseases, National Clinical Research Center for Oral Diseases, West China Hospital of Stomatology, Sichuan University, Chengdu, PR China. Performed the experiments in partial fulfillment of requirements for a degree, statistical analysis, wrote the manuscript.; b Postgraduate Student, State Key Laboratory of Oral Diseases, National Clinical Research Center for Oral Diseases, West China Hospital of Stomatology, Sichuan University, Chengdu, China. Performed statistical evaluation, XRD analysis and proofread the manuscript.; c Postgraduate Student, State Key Laboratory of Oral Diseases, National Clinical Research Center for Oral Diseases, West China Hospital of Stomatology, Sichuan University, Chengdu, China. Contributed substantially to discussion, proofread the manuscript.; d Doctoral Student, Department of stomatology, Chengdu second people’s hospital, Chengdu, China. Performed specimen preparation and shear bond test.; e Postgraduate Student, State Key Laboratory of Oral Diseases, National Clinical Research Center for Oral Diseases, West China Hospital of Stomatology, Sichuan University, Chengdu, China. Performed the optical microscopic and SEM observation.; f Professor, Department of Prosthodontics, State Key Laboratory of Oral Diseases, National Clinical Research Center for Oral Diseases, West China Hospital of Stomatology, Sichuan University, Chengdu, China. Contributed substantially to discussion, proofread the manuscript.; g Associate Professor, State Key Laboratory of Oral Diseases, National Clinical Research Center for Oral Diseases, West China Hospital of Stomatology, Sichuan University, Chengdu, China. Idea, hypothesis, experimental design and proofread the manuscript.; * These two authors contributed equally to this work.

**Keywords:** ceramic-coated zirconia, etching, shear bond strength, surface roughness

## Abstract

**Purpose:**

To evaluate the effects of different hydroﬂuoric acid (HF) concentrations and etching times on the surface topography, roughness, and resin bond strength to ceramic-coated zirconia (CC), and to compare them with the effects of alumina air-abrasion combined with 10-MDP (AA).

**Materials and Methods:**

AA and CC specimens were divided into 12 groups (N = 10). The CC groups were etched with HF at different concentrations (5% or 9.5%) for various durations (0 min, 1 min, 2 min, 3 min, 5 min or 10 min). The surface morphology was analyzed using SEM. Energy-dispersive x-ray spectroscopy (EDS) and x-ray diffraction (XRD) were performed for chemical and crystalline-phase analyses. Surface roughness (Ra) and shear bond strength (SBS) were recorded and statistically analyzed.

**Results:**

The SBS of CC groups initially increased, but then decreased with etching time for both HF acid concentrations. The 9.5% HF group displayed more marked topographical changes and higher Ra compared with the 5% HF group for the same etching period. Mean SBS was lower in the AA group compared with the CC groups etched with 5% HF for 2–10 min and 9.5% HF for 1–3 min (p < 0.05).

**Conclusions:**

Different HF concentrations and etching times influenced the surface topography, roughness, and resin bond strength of/to ceramic-coated zirconia. Etching with 5% HF for 5 min and with 9.5% HF for 2 min, respectively, provided the highest SBS.

In dentistry, the use of yttria-stabilized tetragonal zirconia polycrystal (3Y-TZP) has become increasingly popular.^7^ 3Y-TZP has excellent mechanical strength. However, high opacity hinders its wider application.^3,17^ In order to solve this problem, highly translucent zirconia^14,45^ and multi-layered zirconia^16,19^ have been developed by increasing the content of the stabilizer yttria in zirconia. Compared with conventional zirconia (3Y-TZP), elevated contents of yttria and cubic zirconia crystals make 5 mol% yttria partially stabilized zirconia (5Y-PSZ) more translucent and applicable in more clinical scenarios, especially in the anterior esthetic zone.^38,47^

However, the effects of alumina air-abrasion on the mechanical properties and long-term stability of zirconia remain controversial.^27^ Especially for 5-PSZ, owing to the lack of phase transformation, the zirconia cannot prevent the propagation of cracks caused by sandblasting, resulting in lower flexural and bond strengths.^28^ Moreover, studies have reported that 10-MDP-containing primers cannot maintain stable bond strength after aging due to the hydrolysis of the coordinate bond between ZrO_2_ and 10-MDP.^5,44^

A new method has recently shown very promising results in improving the bond strength of composite cements to zirconia ceramics, even after aging.^24^ It is a vitrification process, which means that low-fusing glasses or ceramic liners adhere to zirconia. The glass layer can be chemically bonded through the silane coupling agent and micromechanically interact with the composite cement more easily after HF acid etching. For silica-based ceramics, the combined use of HF acid etching and silane-coupling agent has been considered the gold-standard pretreatment.^40^ The method is also applicable to silica-coated zirconia and results in high shear bond strength (SBS).^4^ Since this pretreatment method was developed, different etching schemes have been used. However, the optimum HF-acid concentration and etching time for the pretreatment of ceramic-coated zirconia restorations are undefined.^24,37,39^

Several previous in vitro studies have studied diverse HF acid concentrations and etching durations for ceramic-coated zirconia, including 5% for 20 s, 60 s or 120 s; 10% for 20 s, 30 s, or 60 s; and 9.5% for 60 s or 90 s.^4,6,10,12,25,39^ Many studies pretreated ceramic-coated zirconia with 9.5% HF acid etching for 60 s, yielding higher bond strength compared with the sandblasting method.^13,42^ There are a few reports^30,39^ centered on the effects of increasing or decreasing concentrations and durations on the surface morphology, roughness, and bonding characteristics of materials. None of these studies considered whether high acid concentration or long etching time would impair the glass layer of the ceramic-coated zirconia, which has a bilayer structure, since small changes in the glass layer may have substantial effects on its bonding properties.

Consequently, the aim of this study was to evaluate the effects of different HF acid concentrations and varied etching times on the SBS of highly translucent ceramic-coated zirconia with composite resin. Moreover, the morphology of ceramic-coated zirconia surfaces resulting from diverse etching methods was investigated, as were the failure modes of fractured specimens. The results were compared with those of zirconia treated with alumina air-abrasion and 10-MDP.

The null hypotheses were: (1) there is no statistically significant difference in bond strength between the alumina air-abrasion/10-MDP and ceramic coating groups, and (2) different etching times and concentrations would not impact the bond strength of ceramic-coated 5Y-PSZ.

## Materials and Methods

### Specimen Preparation and Experimental Groups

**Fig 1:**
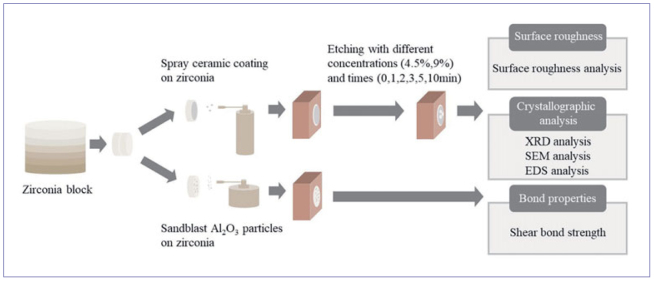
Experimental design.

The materials used in this study are listed in Table 1. The study design is schematically explained in Fig 1. 5Y-PSZ ceramic blocks (Multilayer 3D Pro, Aidite Technology; Qinhuangdao, China) were prepared using a cutting machine (AMD-500, Aidite Technology). The zirconia blocks were sequentially polished with 800- and 1200-grit silicon carbide abrasive papers (Struers; Copenhagen, Denmark) and then sintered according to the manufacturer’s instructions.

**Table 1:** Materials used in this study

Material	Manufacturer	Main composition	Batch No.
Multilayer 3D Pro	Aidite (Qinhuangdao) Technology; Qinhuangdao, China	< 90.67 wt% ZrO_2_, 9.28 wt% Y_2_O_3_, 0.05 wt% Al_2_O_3_	W201072ATA2M-05-P
Variolink Esthetic DC	Ivoclar Vivadent; Schaan, Liechtenstein	Base paste: ytterbium trifluoride, urethane dimethacrylate, 1,10-decandiol dimethacrylate, acetyl-2-thiourea Catalyst paste: ytterbium trifluoride, urethane dimethacrylate, 1,10-decandiol dimethacrylate, α,α-dimethylbenzyl hydroperoxide	Z0054H
Biomic Lisi Fusion system	Aidite (Qinhuangdao) Technology	SiO_2_, Al_2_O_3_, B_2_O_3_, LiO, Na_2_O, Nb_2_O_5_, ZrO_2_	20200710
Korox 50	Bego; Bremen, Germany	50-µm alpha corundum (Al_2_O_3_)	18105980816
Filtek Z350	3M Oral Care, St Paul, MN, USA	Bis-GMA, UDMA, PEG-DMA, TEG-DMA, bis-EMA, 78.5 wt% silica ﬁller	NA71224
Z-Prime Plus (Zirconia Primer)	Bisco; Schaumburg, IL, USA	< 10% biphenyl dimethacrylate, < 20% hydroxyethyl dimethacrylate, < 90% ethanol	1900006023
Porcelain Primer	Bisco	Silane with ethanol and acetone	1900007542
Porcelain Etchant	Bisco	Buffered 9.5% hydrofluoric acid	2000003086
IPS ceramic etching gel	Ivoclar Vivadent	≤ 5.0% hydrofluoric acid	Y03912

Bis-GMA: bisphenol A glycidyl methacrylate; UDMA: urethane dimethacrylate; PEG-DMA: poly-(ethylene glycol)-dimethacrylate; TEG-DMA: triethylene glycol-dimethacrylate; bis-EMA: ethoxylated bisphenol A glycidyl methacrylate.

After sintering, the specimens (6-mm diameter x 2.5-mm height) were embedded in a self-curing epoxy resin (Struers) and then ultrasonically cleaned for 10 min each in pure ethanol and distilled water. These blocks were randomly divided into 12 groups according to the surface conditioning methods listed in Table 2: alumina air-abrasion group (AA) and ceramic coating groups (CC). The CC groups were etched with HF acid at various concentrations for different times. Calculation indicated that 10 specimens per subgroup would provide a power >80%.

**Table 2:** Description of experimental groups

Etching concentration	–	–	5%					9.5%				
Etching time(min)	–	0	1	2	3	5	10	1	2	3	5	10
Code	AA	C0	C_5_1	C_5_2	C_5_3	C_5_5	C_5_10	C_9_1	C_9_2	C_9_3	C_9_5	C_9_10

In the AA group (n = 10), the surfaces of Y-PSZ blocks were sandblasted with 50-μm Al_2_O_3_ particles (Korox 50, Bego; Bremen, Germany) at 0.2 MPa for 20 s perpendicular to the surface at a distance of 10 mm with a sandblaster (Easyblast model, Bego).^2,46^ In the CC groups, a thin layer of ceramic coating (Biomic Lisi, Aidite Technology) was sprayed twice at a distance of 10 cm on the bonding surfaces of the specimens to achieve a homogeneous layer. After spraying, specimens were fired in a ceramic furnace (AUSTROMAT 654 press-i-dent, DEKEMA; Freilassing, Germany), following the manufacturer’s guidelines. After cooling, the ceramic-coated surfaces underwent ultrasonic washing for 10 min each in distilled water and pure alcohol.

Then, 5% hydrofluoric acid gel (IPS ceramic etching gel, Ivoclar Vivadent) and 9.5% hydrofluoric acid gel (Bisco; Schaumburg, IL, USA) were applied as described in Table 2. Group C0 did not receive acid etching. Groups C_5_1 to C_5_10 were etched for 1, 2, 3, 5 and 10 min with 5% HF, respectively. Groups C_9_1 to C_9_10 were etched for 1, 2, 3, 5 and 10 min with 9.5% HF, respectively.

One hundred twenty (120) composite resin cylinders (3-mm diameter x 3-mm height) (Filtek Z350, 3M Oral Care; St Paul, MN, USA) were prepared. All composite cylinders were made as previously described elsewhere.^48^

### X-ray Diffraction (XRD)

The surfaces of as-sintered zirconia, sandblasted zirconia, and ceramic-coated zirconia (without HF acid etching) ceramic blocks were examined by x-ray diffraction (XRD, Empyrean, PANaly; Almelo, The Netherlands) to characterize the phase structure. Cu-K radiation at 40 mA and 40 kV was used to collect XRD data. Spectra were recorded in a 2-theta range from 10 degrees to 90 degrees with a 0.026-degree step. Rietveld analysis was used to quantify the tetragonal (t) and cubic (c) phases of zirconia using GSAS&EXPGUI software (GSAS, Los Alamos National Laboratory, Los Alamos, NM, USA and EXPGUI, Gaithersburg, MD, USA.^41^

### Surface Roughness Assessment

After sandblasting in the AA group and etching in the CC groups, all zirconia samples were ultrasonically cleaned with distilled water for 5 min. Then, the specimens were scanned using white light interferometry (Rtec Instruments; San Francisco, CA, USA) to obtain 3D surface morphology. Average surface roughness (Ra) values were measured with observation software (Gwyddin 2.30, Czech Metrology Institute; Brno, Czech Republic). The method was performed as previously reported.^48^

### Thickness Assessment

Ten specimens after ceramic-coating treatment were embedded in a self-curing epoxy resin base, leaving the untreated surface exposed. They were ground under water-cooling in cross sections from one side to the center of the zirconia with a polishing machine (Tegramin; Struers). Then, the samples were polished using MD-Dac diamond suspension and a DiaPro Dac polishing cloth (Struers) of 3 μm to acquire a mirror-like surface. The cross-sectional surface was observed using SEM so that the glaze thickness could be measured.

### Bonding Procedure

In the AA group, a thin layer of Z-Prime Plus (Bisco) was uniformly applied. After 20 s, the primed blocks were dried gently with oil-free air for 15 s.^44^ In the CC group, after etching and cleaning, a thin layer of a silane primer (Porcelain Primer, Bisco) was applied onto the surface. Finally, the specimens were gently dried with oil-free air for 20 s.

Variolink Esthetic DC cement (Ivoclar Vivadent; Schaan, Liechtenstein) was then mixed and used to bond the composite resin cylinders onto treated zirconia surfaces following the manufacturer’s instructions. All excess cement was removed with a microbrush, and the margins were light cured for 20 s per surface (800 mW/cm^2^, Bluephase N, Ivoclar Vivadent). The cement was cured under a sustained load of 0.5 kg for 5 min to ensure its absolute setting.^1^ After bonding, the samples were stored in distilled water for 24 h at 37°C.

### Shear Bond Strength (SBS) Test

After 24-h storage, the specimens were subjected to shear bond strength testing in a universal testing machine (Instron Model 5565; Norwood, MA, USA) at a crosshead speed of 0.5 mm/min.^32,36^ Until debonding occurred, a chisel-shaped device applied the load to the surface at the junction of composite cement and zirconia. SBS (MPa) was subsequently measured using Bluehill universal software (Instron). The bond strength was calculated as follows: SBS = F/A, where F = maximum force (N) and A = bonding area (mm^2^).

### Fractographic Analysis

After the SBS test, the fractured specimen surfaces were observed under a light microscope (LM, BX51RF, Olympus; Tokyo, Japan). Failure was classified as described below.^39^

- AA group: cohesive failure (C): more than 80% failure within the zirconia substrate or composite cement; adhesive failure (A), more than 80% failure at the zirconia/resin interface; mixed failure (M), mixed cohesive and adhesive failure modes.
- CC group: cohesive failure (C): more than 80% of failure within zirconia substrate, composite cement, or ceramic coating layer; adhesive failure (A): more than 80% of failure at the resin/ceramic coating interface or ceramic coating/zirconia interface; mixed failure (M): mixed cohesive and adhesive failure modes.

### Surface Morphology and Elemental Analysis

Three samples from each group were assessed using scanning electron microscopy (SEM, JSM-IT500, JEOL; Tokyo, Japan) to evaluate the microstructure and surface topography. After treatment, all specimens were coated with gold (DII-29010 sCTR DII-29010 sCTR Smart Coater, JEOL) and observed using SEM at 10 kV. The elemental compositions of as-sintered zirconia, sandblasted zirconia, and ceramic-coated zirconia (with and without HF acid etching) were analyzed by energy-dispersive x-ray spectroscopy (EDS). EDS was also used on the cross-sectional surface of as-sintered zirconia to analyze changes in the elemental distribution by line-scan analysis.

### Statistical Analysis

Data were statistically analyzed using SPSS 20.0 (IBM; Armonk, NY, USA). The Kolmogorov-Smirnov test was conducted to assess whether variables were normally distributed. SBS data were analyzed by two-way ANOVA (independent variables, HF acid concentration and etching time; dependent variable, SBS) followed by one-way ANOVA and the post-hoc Dunnett-T3 test.^33,39,43^ One-way ANOVA was performed to analyze Ra data, followed by multiple comparison between the groups using the Dunnett-T3 test. The significance level was set at α = 0.05.

## Results

### Surface Topography and Elemental Compositions

**Fig 2:**
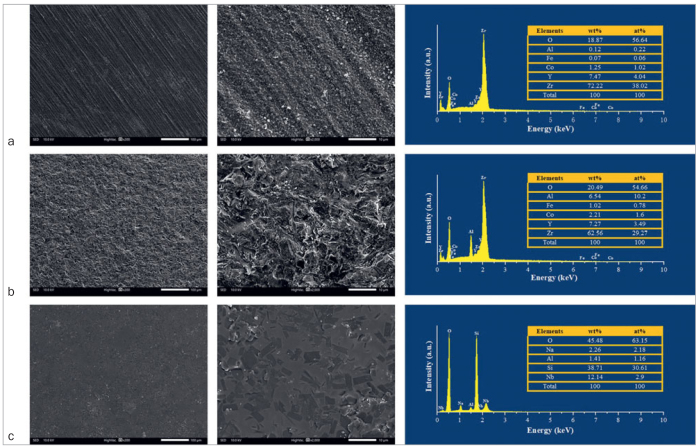
Representative SEM images at low (200X) and high (2000X) magniﬁcation: (a) as-sintered zirconia: regular scratches and typical grains. (b) Sandblasted zirconia with 50-µm Al_2_O_3_ particles for 20 s: rough surface with sharp edges and grooves. (c) Ceramic-coated zirconia: homogenous ceramic layer covered with needle-like crystals. The third image column shows the corresponding EDS analysis of elemental composition on zirconia surfaces: Zr, zirconium; Al, aluminium; O, oxygen; Y, Yttrium; Fe, iron; Si, silicon; Nb, niobium; Na, sodium.

Representative SEM images and EDS of as-sintered zirconia, sandblasted zirconia, and ceramic-coated zirconia are shown in Fig 2. Figures 2a and 2b show typical zirconia grains, while Fig 2c displays a homogeneous glass layer on the zirconia surface. EDS analysis revealed the presence of zirconium (Zr), aluminum (Al), and oxygen (O) in as-sintered and sandblasted zirconia specimens. In contrast, silicon (Si), niobium (Nb), and sodium (Na) were detected on the surface of ceramic-coated samples.

**Fig 3:**
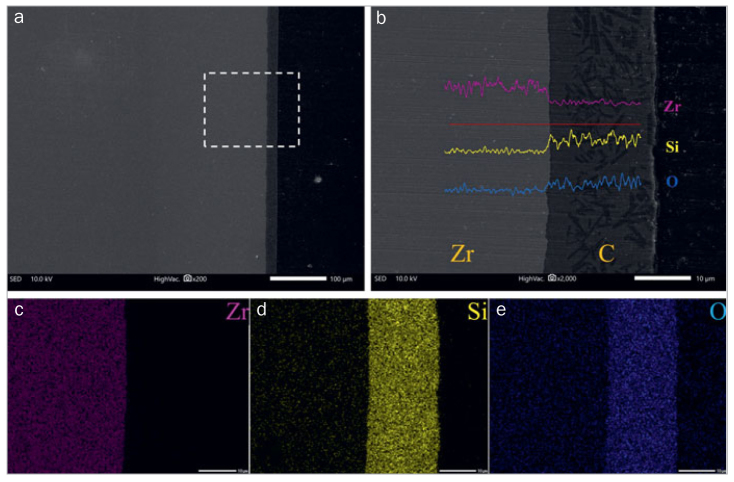
Cross-sectional view of a zirconia disk with 2 layers of ceramic coating. (a) SEM image (original magnification 200X). (b) Line scan analysis and thickness measurement of the interfacial zone of zirconia and the ceramic coating (original magnification 2000X). (c–e) Mapping analysis of elements: O, oxygen; Zr, zirconium; Si, silicon.

Typical SEM images of the cross sections of ceramic-coated zirconia are shown in Fig 3. A homogeneous, gap-free glass layer with no pits or defects covered the zirconia substrate in the cross-section. The measured average cross-sectional coating thickness was 17.0±0.7 µm. A line-scan analysis was further performed to examine elemental composition changes along the line based on ion diffusion. Si and Zr diffused at the interface between the zirconia and the ceramic liner.

**Fig 4:**
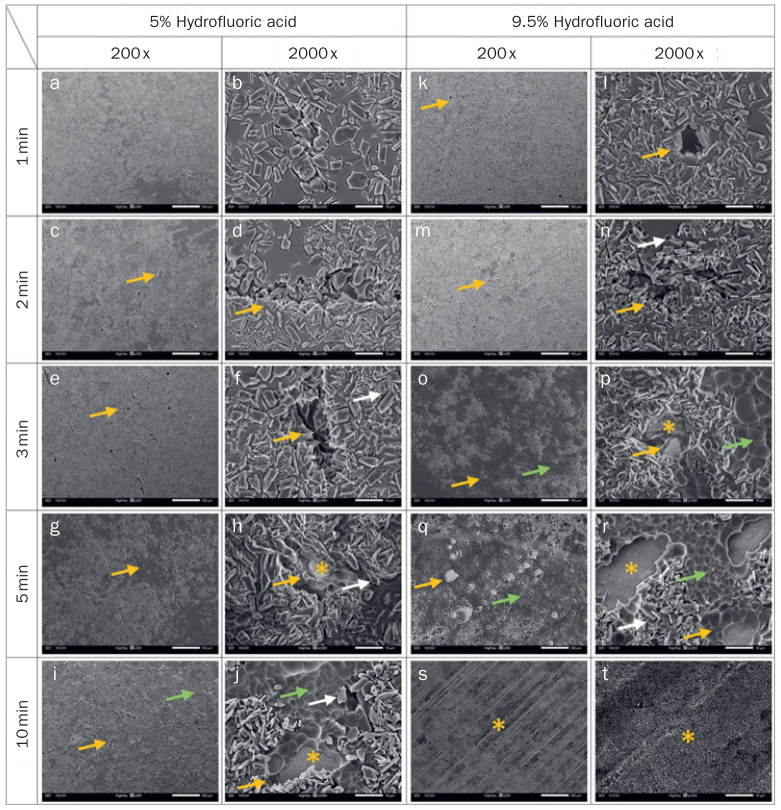
Representative SEM micrographs of 5-PSZ surface etched with 5% HF and 9.5% HF. Mild dissolution of a thin glass-phase layer and exposed crystals are shown for 5% HF in a and b. Moderate glass-phase dissolution is observed for 5% HF in c–f and for 9.5% HF in k–n. More glass phase is dissolved leaving crystals (white arrows) exposed and resulting in more numerous, larger and deeper pores and grooves (yellow arrows). Extensive glass-phase dissolution is evident. The time- and concentration-dependent drastic dissolution of glass phase is apparent for 5% HF in g–j and for 9.5% HF in o–r. Some unsupported crystals fell out, leaving numerous crater-like cavities (green arrows) on the surface of the glass layer. Some layers even collapsed to expose the zirconia base (yellow *). The glass layer was nearly consumed and the zirconia base was exposed when 9.5% HF was applied (s–t).

**Fig 5:**
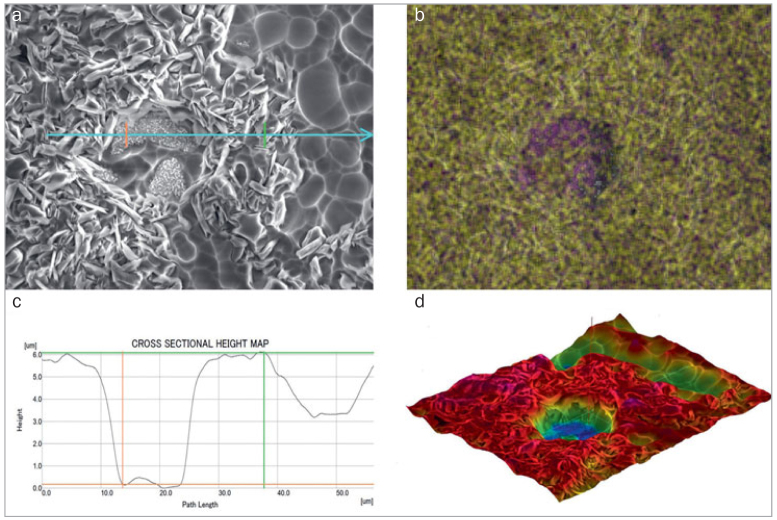
Etched surface of Fig 4p. (a) SEM image (original magnification 2000X). (b) Corresponding EDS mapping. The yellow area represents Si on the zirconia surface; the purple area represents exposed zirconia after etching. (c) Blue line represents depth proﬁles. (d) Reconstructed three-dimensional map of the etched surface.

SEM examinations of ceramic-coated zirconia after HF acid etching with different concentrations and etching times are shown in Fig 4. HF concentrations and etching times directly influenced the etching morphology. As etching time increased, the surface became increasingly rough due to glass-phase dissolution. More micropores of different shapes and sizes were found on the ceramic layer, with some even as deep as the zirconia layer. The 9.5% HF acid etching pattern (Figs 4k to 4t) appeared to be more pronounced than the 5% HF acid etching pattern (Figs 4a to 4j). When etching time was too long (Figs 4s and 4 t), the ceramic layer was nearly completely dissolved, and the zirconia base was exposed. Treated surface morphology (SEM, 2000X) and its corresponding 3D topography, depth profiles, and EDS map with 9.5% HF etching for 3 min are shown in Fig 5. The etched surface was rough. The exposed zirconia layer exhibited the greatest depth, followed by pore areas produced by pulled-out crystal grains, and an area including crystal grains.

### XRD Findings

**Fig 6:**
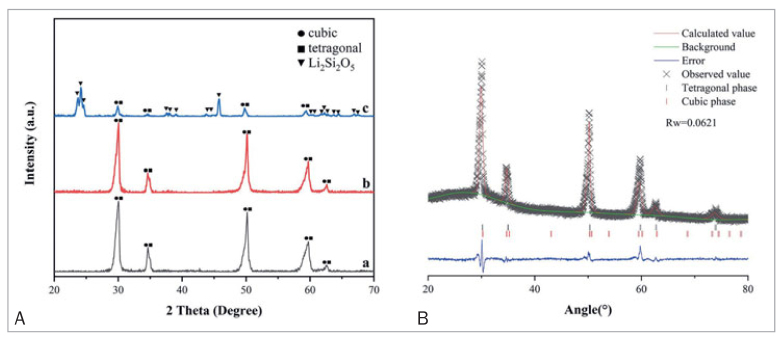
(A) XRD patterns of the sintered zirconia (a), sandblasted zirconia (b), and ceramic-coated zirconia (c). The representative peaks of tetragonal zirconia, cubic zirconia, and Li_2_Si_2_O_5_ phase are marked. (B) Rietveld fitting refinement results of as-sintered zirconia.

The XRD patterns of as-sintered zirconia (a), sandblasted zirconia (b) and ceramic-coated zirconia (c) are shown in Fig 6A. The peaks in accordance with tetragonal (t-ZrO_2_) and cubic phase (c-ZrO_2_) zirconia were detected in the diffraction pattern of as-sintered zirconia, sandblasted zirconia, and ceramic-coated zirconia. The monoclinic phase (m-ZrO_2_) was not detected in any of the zirconia ceramics. Diffraction peaks of Li_2_Si_2_O_5_ were observed in the XRD pattern of ceramic-coated zirconia. Rietveld analyses of the XRD patterns showed that this 5 mol% yttria-stabilized zirconia ceramic contained approximately 87.40% c-ZrO_2_ phase and 12.60% t-ZrO_2_ phase.

### Surface Roughness Analysis

**Fig 7:**
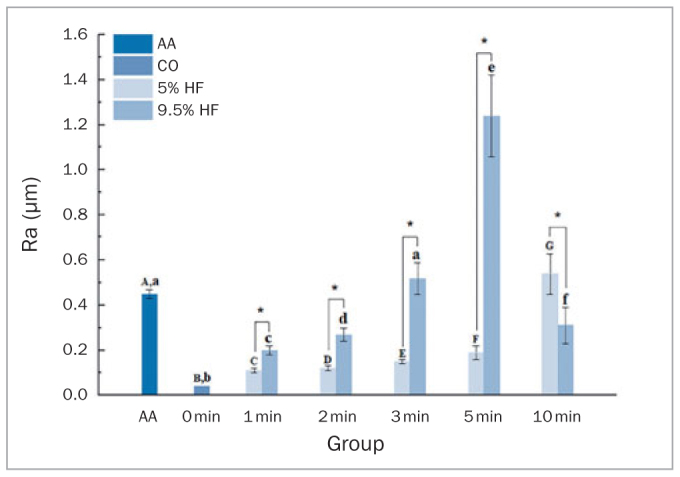
Ra after different surface treatments. Different lowercase letters indicate significant differences between groups with the same concentration (p < 0.05). *Significant differences between groups for the same etching time.

Ra means and standard deviation (SD) of the zirconia surface after different surface pretreatments are shown in Fig 7. Mean Ra values were higher in the AA group compared with the CC groups, except for the C_5_10, C_9_3, and C_9_5 groups (p < 0.05).

In the CC groups, the Ra values varied with HF acid concentrations (p < 0.001) and etching duration (p < 0.001). Apart from the C_9_10 group, 9.5% HF acid-etched specimens exhibited higher Ra than those subjected to 5% HF acid for the same etching duration (p < 0.05). It was apparent that the Ra values initially increased and subsequently decreased with increasing etching duration when using 9.5% HF, but they continuously increased over the whole etching duration with 5% HF. The highest Ra values were found in C_5_10 and C_9_5.

### Shear Bond Strength

**Fig 8:**
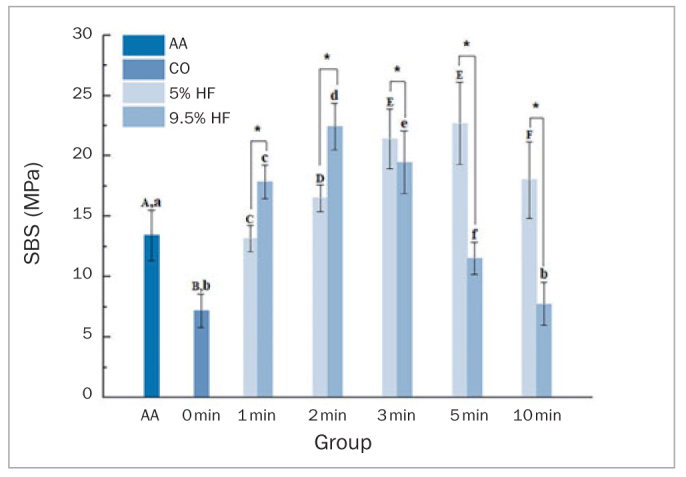
The SBS after different surface treatments. Different lower-case letters indicate significant differences between groups with the same etching concentration (p < 0.05). *Significant differences between groups for the same etching time.

HF acid concentration (p = 0.004) and etching time (p = 0.000) directly affected SBS (Fig 8). The mean SBS was lower in the AA group than in the CC groups etched with 5% HF for 2 to 10 min, and with 9.5% HF for 1 to 3 min (p < 0.05).

With a 5% HF-acid concentration, SBS significantly increased as etching time was prolonged from 0 to 5 min (p < 0.05), but no statistically significant differences were found between the C53 and C55 groups. At an acid-etching duration of 10 min, SBS decreased significantly (p < 0.05).

With 9.5% HF acid, SBS was significantly higher in the 2-min group compared with the 1-min group (p < 0.05). When the etching duration increased from 3 to 10 min, SBSs decreased significantly (p < 0.05). No statistically significant differences were found between etching for 0 and 10 min (p > 0.05).

### Fractographic Analysis

**Fig 9:**
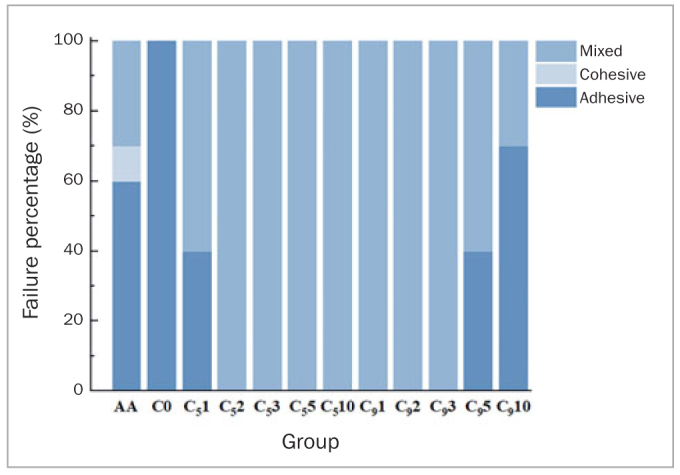
Distribution of failure modes among groups after SBS testing.

The failure mode percentages are shown in Fig 9. When the specimens were etched with 5% HF for 2 to 10 min and with 9.5% HF for 1 to 3 min, they exhibited mixed failure. All specimens in the C0 group presented adhesive failure. Other CC groups, including C_5_1, C_9_5, and C_9_5, showed either adhesive or mixed failure. All three failure modes were present in group AA (adhesive, cohesive, and mixed).

**Fig 10:**
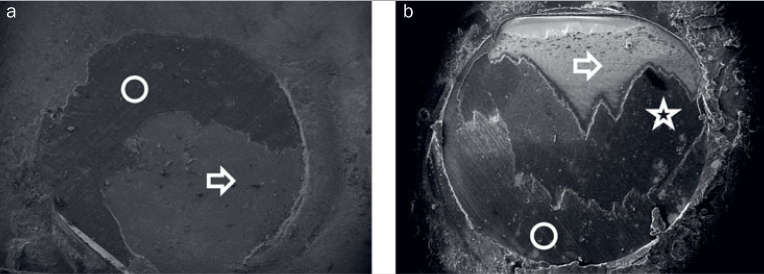
Representative mixed failure mode of AA group (a) and CC group (b) specimens under SEM. Star: ceramic-coated surface; circle: remaining composite cement; arrow: exposed zirconia.

Representative SEM images of mixed failure modes from the AA (A) and CC groups (B) are shown in Fig 10. The boundary between the fused glass layer and the composite cement, as well as between the fused glass layer and zirconia, were clearly demarcated from each other. In the AA group, the boundary was between zirconia and composite cement.

## Discussion

The results of this study revealed that the CC groups had significantly higher SBS compared than did the AA group at etching times ranging from 2 to 10 min for 5% HF and from 1 to 3 min for 9.5% HF (p < 0.05). Thus, the first null hypothesis was rejected. Moreover, the results indicated that various acid etching methods influenced the microstructural morphology, Ra, and SBS of/to ceramic-coated zirconia. Consequently, the second null hypothesis was also rejected.

Although SBS testing has been criticized for the development of inhomogeneous stress distributions at the adhesive interface,^15,30^ the method was chosen in this study for several reasons. First, the relatively high hardness of zirconia makes sectioning of sintered specimens very difficult, complicating the production of samples for micro-bond tests. The macroshear test is easy to perform and does not damage the samples during production. Secondly, SBS is the most commonly used test methodology.^15,30^ SBS data showed the smallest standard deviations and specimen preparation did not yield pre-test failures, compared with other bond testing methods.^15,24^ Thirdly, considering that the main purpose of this work was to assess the effect of hydrofluoric acid etching on the bond strength of ceramic-coated zirconia, any test design (“micro” or “macro”) would have allowed comparing differences between groups.^31^

The novel method of ceramic coating combined with HF acid etching and silane coupling can improve bond strength, and has recently been widely accepted.^13,22,43^ In this study, glass coating was applied onto the intaglio surface of the zirconia substrate to create a homogeneous layer without pits or defects. Needle-like crystals embedded in the glass phase were observed (Fig 2c). EDS analysis confirmed that the surface was rich in Si. XRD results further showed that the main crystallized phase was Li_2_Si_2_O_5_. A chemical siloxane network was created on the zirconia surface. When the silica-based layer was HF acid etched, the glass phase (SiO_2_) was selectively dissolved by the acid, exposing the crystalline structure. Acid etching roughened the glass surface, enhancing micromechanical interlocking retention and helping resin tags infiltrate deeply into the pores of the ceramic coating.^22,35^ Moreover, the silane-coupling agent applied on the etched, roughened silica-rich surface increased the chemical bond strength between the resin composite and ceramic-coated zirconia.^9,40^

However, Ra and SBS may change with etching duration. According to the above results, the etching process of ceramic-coated zirconia could be divided into three stages. At the first etching stage, the CC groups treated with 5% HF yielded lower immediate SBS compared with the control (AA) group at etching durations ranging from 0 to 1 min. The lowest SBS and Ra were found in specimens without HF acid etching treatment. The unetched surface only demonstrated chemical adhesion, without micromechanical interlocking retention, which resulted in a high bond-failure rate between 5-PSZ and composite cements. This suggests that chemical treatment of the surface alone cannot mediate stable bond strength to the ceramic-coated zirconia.^20,26^ When the specimens were etched for 1 min, poor dissolution of the glass phase was observed, and the exposure of crystals was limited to the surface layer. SBS was relatively low, mainly because the glass was not etched sufficiently within a short period of time. Due to the lower Ra, the glass layer could not provide sufficient microinterlocking via resin tags. The adhesion was brittle between cement and glass layers, and fractures tended to occur between the interfaces. The results are consistent with previous investigations,^10,39^ showing that short etching durations could not produce good bonding quality to ceramic-coated zirconia.

At the second etching stage, when the etching time ranged from 2 to 5 min for 5% HF, more glass was dissolved and more crystals were exposed at various depths. The SBS increased with increasing Ra. The characteristic honeycomb-like surface extended the bonding surface area and facilitated resin tag infiltration into the ceramic porosities to create favorable micromechanical interlocking retention.^13,35^ Moreover, the rough silica-based surface provided a better chemical environment for the ceramic to react with the silane-coupling agent. The CC groups displayed signiﬁcantly higher SBS compared with the control AA group at this etching duration.

As the etching time was prolonged from 5 to 10 min for 5% HF at the third stage, Ra increased, while SBS decreased. Even so, SBS was higher in the 5% HF group after 5-min etching compared with the AA group. During this period, some crystal grains were pulled out, leaving pores on the glass surface^29,34^ due to substantial and irregular loss of the surrounding glass phase. Lengthy HF acid etching even caused surface ceramic layer collapse and zirconia surface exposure. On the one hand, larger, more numerous pores might impair mechanical interlocking sites.^34^ On the other hand, the exposure of zirconia might weaken the effect of the chemical siloxane network. Lengthy HF acid etching might have a harmful influence on the SBS to ceramic-coated zirconia. Therefore, we believe that the bond strength of ceramic-coated zirconia after HF acid etching is determined by the balance between micromechanical interlocking retention and chemical bonding.

C_5_2, C_5_3, C55, and C_5_10 showed 100% mixed failures and had higher SBS compared with the AA group, in agreement with other studies.^10,11,21,25^ It suggested that siloxane bonding at the interface between the composite cement and ceramic-coated zirconia provides stable retention, as does the combination between the ceramic layer and zirconia. However, little is known about the interaction between zirconia and the fused ceramic glaze layer. One study^8^ considered that the adhesion between the glass and zirconia relied only on van der Waals and electrostatic forces. Recently, several studies have observed that some components of silica-containing glass-ceramic liner materials, such as Al, Na, Si, and K, may diffuse into the surface of zirconia, creating chemical bonding between zirconia and the fusion glass-ceramic coating.^18,21^ The present study also observed Si diffusion from the ceramic layer into the zirconia substrate, which created a reliable bond between two materials. Overall, the adhesion between the glass and zirconia is excellent, and the failure modes are in line with the results of bond strength testing for the CC group etched with 5% HF.

Likewise, ceramic-coated zirconia etched with 9.5% HF showed the same SBS and etching performance trend as did groups treated with 5% HF. The 9.5% HF acid etching pattern appeared to be more pronounced than that resulting from 5% HF acid etching. Elevated HF acid concentration causes more ionized HF to react with SiO_2_ from the glass phase.^33^ Thus, crystals from the glassy phase were easier to expose, and deeper, larger pits were created when specimens were treated with 9.5% HF compared with 5% HF for the same duration. Specimens treated with 9.5% HF could enter the second stage faster than those etched with 5% HF. When the etching duration reached 1 min, SBS was higher in the 9.5% HF group compared with the AA group, while the 5% HF group had a lower value than did the AA group. Other studies also used similar parameters and obtained comparable results.^13,43^ The ceramic-coated zirconia etched with 5% HF for 5 min showed the highest SBS, while 9.5% HF etching for 2 min could lead to the highest SBS. Ceramic-coated zirconia etched with HF acid at a higher concentration would reach the peak values of SBS and Ra values in a shorter time. At the third etching stage, where the etching duration reached 5 min for 9.5% HF, the specimens showed the highest Ra but lower SBS. The glass layer was gradually dissolved, and the zirconia base was exposed. The chemical siloxane network constructed on the surface was nearly ineffective. In addition, zirconia suffered from adhesive failures with lower bond strength.

In summary, surface morphology analysis and SBS data showed HF-acid concentration and duration have a significant influence on ceramic-coated zirconia. When the appropriate etching scheme was applied, the surface modification of low-fusing glass would result in higher SBS compared with the conventional method, ie, alumina air-abrasion combined with 10-MDP-containing primer. Considering the potential clinical risks of lengthy acid etching, etching conditions of 2 to 5 min for 5% HF and 1 to 2 min for 9.5% HF can be recommended.

The low-fusing glass method described here seems to present a suitable pretreatment option for resin bonding to 5-PSZ. It must be mentioned that the shapes and sizes of the specimens tested here do not resemble clinical restorations. Future investigations should evaluate the effects of this treatment on the mechanical properties and the internal and marginal fit of prostheses, which can help determine the overall adaptability of a prosthesis to tooth-like preparations. Standardizing the clinical procedure and realizing controlled application of the ceramic-coating layer also deserves further attention. Moreover, the bond strength could have been influenced by long-term water storage or aging with thermocycling.^18^ Further studies are also required to determine the effects of different acid etching modes on bond durability and mechanical strength of ceramic-coated zirconia. The effects of different brands of zirconia, ceramic liner, silane, and composite cements on SBS should also be investigated.

## Conclusion

The etching scheme of highly translucent ceramic-coated zirconia affected the mean SBS. Increases in HF-acid concentration and etching duration significantly altered the surface microstructure and roughness of highly translucent ceramic-coated zirconia. The effect of HF acid etching on the bonding performance of ceramic-coated zirconia is concentration- and time-dependent. Insufficient etching time and HF concentration does not provide stable bond strength. Lengthy HF acid etching might have a harmful effect on SBS. Pre-treatment by acid etching with 9.5% HF for 2 min and 5% HF for 5 min resulted in the highest bond strengths, not only for the ceramic-coating method, but also for the conventional method, ie, alumina air-abrasion combined with 10-MDP-containing primer.

## Clinical relevance:

HF concentration and etching duration should be chosen carefully for optimal clinical bonding when using ceramic-coated 5-PSZ.
